# Seeing and believing: recent advances in imaging cell-cell interactions

**DOI:** 10.12688/f1000research.6435.1

**Published:** 2015-07-17

**Authors:** Alpha S. Yap, Magdalene Michael, Robert G. Parton

**Affiliations:** 1 Division of Cell Biology and Molecular Medicine, Institute for Molecular Bioscience, The University of Queensland, St. Lucia, Brisbane, Queensland, 4072, Australia

**Keywords:** cell-cell interactions, cadherin, adherin junctions, cell-cell junctions, imaging, electron microscopy

## Abstract

Advances in cell and developmental biology have often been closely linked to advances in our ability to visualize structure and function at many length and time scales. In this review, we discuss how new imaging technologies and new reagents have provided novel insights into the biology of cadherin-based cell-cell junctions. We focus on three developments: the application of super-resolution optical technologies to characterize the nanoscale organization of cadherins at cell-cell contacts, new approaches to interrogate the mechanical forces that act upon junctions, and advances in electron microscopy which have the potential to transform our understanding of cell-cell junctions.

## Introduction

Cell biologists are often resolutely visual people: we believe most what we can see best. This is a heritage of the history of our discipline, which found its roots in work such as Palade’s application of electron microscopy to characterize cellular and subcellular structure. Later, the introduction of antibody technologies allowed morphology to be complemented by molecular specificity. Advances in our understanding of cell biology thus have been driven by the combination of new technologies in microscopy and new reagents that allow us to probe cellular constitution and function.

In this article, we aim to review how this combination of new technologies and reagents has advanced our understanding of the biology of cadherin-based adherens junctions. We focus on three of these advances. First, we have come to appreciate that adherens junctions are not homogenous collections of cadherin receptors but rather have patterns of organization that are apparent at the nanoscale (smaller than a micron) and mesoscopic scale (tens of microns). Second, we now know that cadherin-based adhesions are active mechanical agents where cells generate force to test their environment and sense forces that are applied upon them. Third, although many of these insights have come from developments in light microscopy, the last 5 to 10 years have also seen the development of dramatic new tools in electron microscopy; these have yet to be widely applied to study cell-cell interactions, but their potential is enormous.

### Organization and structure of adherens junctions

**Figure 1.**
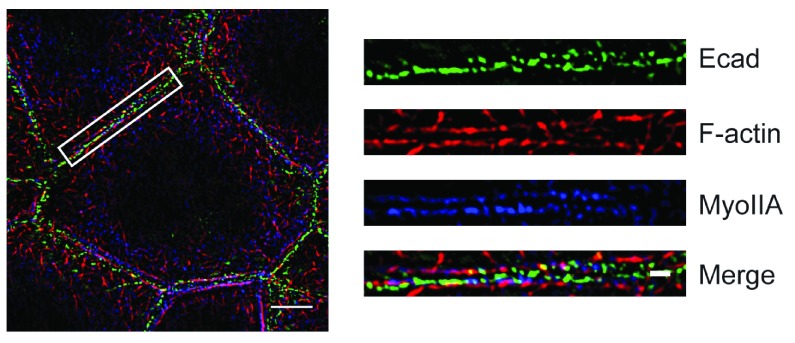
Visualization by structured illumination microscopy of the contractile apparatus at the epithelial zonula adherens. Caco-2 cells were stained for E-cadherin, F-actin, and myosin IIA. Details in the region marked by the box are shown on the right side. Bars = 5 μm (on the left side) and 1 μm (for the magnified images on the right).

Optical microscopy has been revolutionized by techniques that have overcome the limits that the diffraction of light imposes on spatial resolution ^1^. These include approaches such as structured illumination ( Figure 1) and fluorescence photoactivated localization microscopy/stochastic optical reconstruction microscopy (F-PALM/STORM), which are now being applied to the characterization of cell-cell junctions ^2– 4^. Already, they have provided valuable insights into how cadherins are organized into clusters at the nanoscale.

The capacity for cadherins to organize into lateral clusters was observed nearly 20 years ago when it was identified as a mechanism that could strengthen cadherin-based adhesion ^5, 6^, probably by increasing the avidity of adhesive binding between cadherins and their ligands ^6^. However, those experiments were performed by using reductionist models, such as fibroblasts engineered to express E-cadherin ^5^ or cells adherent to substrata coated with C-cadherin ligands (analogous to the two-dimensional substrata that students of integrin biology have long used to study focal adhesions and focal contacts) ^6^. It was more difficult to determine the extent to which lateral clustering might occur at the native cell-cell contacts formed between cells that express endogenous cadherins, such as simple polarized epithelia. High-resolution confocal imaging had identified clustering in Drosophila embryos ^7^ and cultured mammalian cells ^8^ but did not readily permit quantitative analysis of the extent or nature of this clustering. More commonly, cadherins appeared to distribute extensively at contacts between cells, as if junctions represented carpets of homoligated cadherin complexes.

Two recent articles applied PALM/STORM to characterize nanoscale E-cadherin distribution in Drosophila embryonic epithelia ^3^ and cultured mammalian cells ^4^. Both clearly demonstrated that E-cadherin was distributed in polydisperse clusters throughout the junctions of these epithelial systems. They confirm that lateral clustering is a fundamental feature of the supramolecular organization of cadherins at junctions. Furthermore, mammalian junctions displayed clusters with a preferred size of approximately 50–60 nm, which then could organize into larger-scale groups ^4^.

More detailed quantitative analysis also provided provocative insights into the cellular control of clustering. Earlier studies based on analysis of the crystal structure of cadherin ectodomains proposed a model in which *trans*-interactions between the ectodomains presented on the surfaces of neighboring cells, combined with *cis*-interactions between ectodomains on the same cell surface, could cause packing into clusters ^9, 10^. However, the cytoplasmic tail also supports clustering in cells ^4, 11^. Wu *et al.* ^4^ (2015) found that the molecular density of cadherins could vary even within the same cluster. Some regions within clusters showed high packing density, comparable to that predicted from the crystal structures; this required the ability of cadherins to undergo both *cis*- and *trans*-interactions. However, even when the ability to make *cis*- and *trans*-interactions was ablated, cells could still make clusters with a size (50–60 nm) similar to those of wild-type cadherins. This implied that adhesive ligation might not be necessary for clustering to occur. Indeed, clusters were observed at the free surfaces of cells, where cadherins could not engage in adhesion, and even with cadherin mutants that lacked the whole adhesive ectodomain ^4^. Instead, clustering required an intact actin cytoskeleton, and detailed inspection suggested that cadherin clusters might be delimited by “corrals” of cortical actin. Consistent with this, Troung Quang *et al.* ^3^ (2013) demonstrated that F-actin integrity was necessary to stabilize cadherin clusters. Overall, this implies that multiple mechanisms can influence clustering. In one model, cortical actin may define a minimal cadherin cluster, which does not require adhesive ligation; however, the packing of cadherin molecules within clusters is increased upon ligation.

Comparison of the two studies also highlights how the operational definition of “clusters” can fundamentally condition the detailed quantitative analysis and its interpretation. For example, although both groups used the same algorithm to analyze their data, they differed in their definition of clusters and hence in the metrics that they used to describe the clusters. Troung Quang *et al.* ^3^ used a kinetics-based model which defined “size” in stoichiometric terms, as the number of cadherin molecules present within clusters. In contrast, Wu *et al.* took a more empirical approach that focused on the spatial size of the clusters. What emerged with the first approach, as confirmed by Wu *et al.*, was that the distribution of “sizes” followed a power law, implying that the mechanisms that governed how many cadherin molecules accumulate in a cluster did not have a preferred number. However, a power law relationship was not evident when “size” was defined spatially, as the diameter of the cluster, the data being better fit to a Gaussian distribution. Taken together, these findings suggest that there may be a preferred spatial dimension to a cadherin cluster (approximately 50 nm), but within this physical limit the number of cadherin molecules that can be accumulated varies over a wide range. This emphasizes that how the apparently straightforward notion of “size” is explicitly implemented in the computational analysis will deeply influence data interpretation with these approaches.

More generally, these studies suggest that the notion of a “cluster” may need to be conceptually defined with greater precision than we have sometimes done in the past. The work of Wu *et al.* suggests that there may be elemental units that may reflect the spatial organization of the cortical actin cytoskeleton. However, these appear to be able to organize into larger-scale conglomerations and accumulate a variable number of cadherin molecules. It should be remembered that cadherins exist as macromolecular complexes with a range of associated proteins ^10^. So the clusters of cadherins will more likely represent nanoassemblies of many different proteins. What mechanisms define these larger-scale patterns of organization have yet to be established. However, insofar as the phenomenon of receptor clustering has been implicated in regulating cellular processes as fundamental as cell signaling ^12, 13^ and receptor sensitivity ^14^, it will be important for us to clearly specify what aspect of “clustering” we are talking about when we come to further analyze the role that clustering plays in cadherin biology.

### Probing the mechanical properties of cadherin junctions

A fundamental advance in our understanding of cadherin biology has come from the realization that cadherin adhesion serves to couple the contractile cortices of cells together ^15, 16^. Indeed, cadherins may promote the biogenesis of the junctional contractile apparatus itself ^8, 17^. An important part of this advance has come from the application of tools and theory from the physical sciences to biology, combined with the development of new reagents that allow us to measure molecular-scale tension.

For example, one of the most popular approaches to assessing tension is to cut regions (cortices, junctions, and whole cells) with a laser and measure the instantaneous velocity of recoil as an index of the tension that had been present beforehand ^18^. This has been used in embryonic tissues ^19, 20^ as well as in cell culture models ^8^. Similar nanoablation techniques have been combined with physical theory to characterize patterns of cortical tension in *Caenorhabditis elegans* embryos ^21^. It should be noted that, though intuitively attractive, the velocity of recoil is not itself a direct measure of tension. Instead, recoil velocity reflects the ratio of tension over frictional forces. When used to infer tension, this assay assumes that the frictional elements (which would reflect the viscoelastic properties of the junctions) do not change between experimental maneuvers ^18^. Ultimately, precise interpretation of recoil velocity needs to be informed by measurements of junctional viscoelasticity ^22^. Other indirect assays have measured junctional movements to infer tension when combined with explicit mechanical models ^23, 24^.

These essentially mesoscopic measurements can be productively complemented by the use of molecular-level tension-sensitive biosensors, such as the Förster resonance energy transfer (FRET)-based system developed by Grashoff *et al.* ^25^. This sensor reports tension based on the displacement of FRET pairs that are separated by an elastic linker derived from spider silk. The tension sensor (TS) module has been inserted into a range of proteins, where it reported tension over both cadherins (E-cadherin and VE-cadherin ^26, 27^) and vinculin at cell-cell junctions ^28^. Of note, the TS module was calibrated *in vitro*, where it displayed greatest sensitivity over a range of 1–6 pN ^25^. Therefore, its efficacy as a reporter will depend on whether the molecular-level forces that are present fall within its range of sensitivity. Nonetheless, the mesoscopic and molecular-scale approaches to measuring tension are complementary and it is informative to compare both assays, where possible. For example, in mature focal adhesions, which are thought to be sites where contractile force is exerted upon integrin complexes ^29^, vinculin itself can become uncoupled from tension ^25^, despite the integrity of the focal adhesion being unchanged. Thus, molecular-level tension may not always correlate with mesoscopic-level tension.

An important issue for the future is to better characterize the material properties of cell-cell junctions. Until now, we have lacked the tools to measure those properties. But things have begun to change. He *et al.* ^30^ (2014) followed the patterns of flow of microbeads injected into Drosophila embryonic epithelia to assess the patterns of mechanical connectivity between cells. They concluded that lateral cell-cell junctions did not present substantive barriers to hydrodynamic flow between cells. Furthermore, Bambardekar *et al.* ^22^ (2015) demonstrated that it was possible to manipulate cell-cell junctions in Drosophila embryonic epithelium by using optical tweezers and thereby assess the mechanical properties of the junctions. Whether such approaches will be more broadly applicable in other cellular systems remains to be tested.

### New directions in ultrastructural analysis of cell-cell interactions

The suite of light microscopic techniques available to researchers is impressive, but we are also witnessing a revolution in electron microscopy, from high-resolution structural analysis to ultrastructural analysis of whole tissues in three dimensions (3D). Many of these methods are becoming routine in laboratories throughout the world but have not been extensively applied to the study of cell-cell interactions. Here, we will briefly summarise relevant techniques and their possible applications in this area.

Ultrastructural methods can potentially answer how molecular interactions and spatial interactions contribute to the formation and function of junctional assemblies. The ideal method would allow visualization of both the cytoskeleton and membranous elements which together generate the active junctional complex; it should also have the resolution to identify the location of individual protein components in the context of a 3D volume of the cell-cell contact sites. This should include actin and other cytoskeletal networks, cadherin, and actin-binding proteins and should be correlated with real-time observations of junctional dynamics. Although some elements can be recognized by morphology alone (cytoskeleton and junctions), new labeling methods are now facilitating visualization of otherwise undetectable components and can be combined with 3D methods.

**Figure 2.**
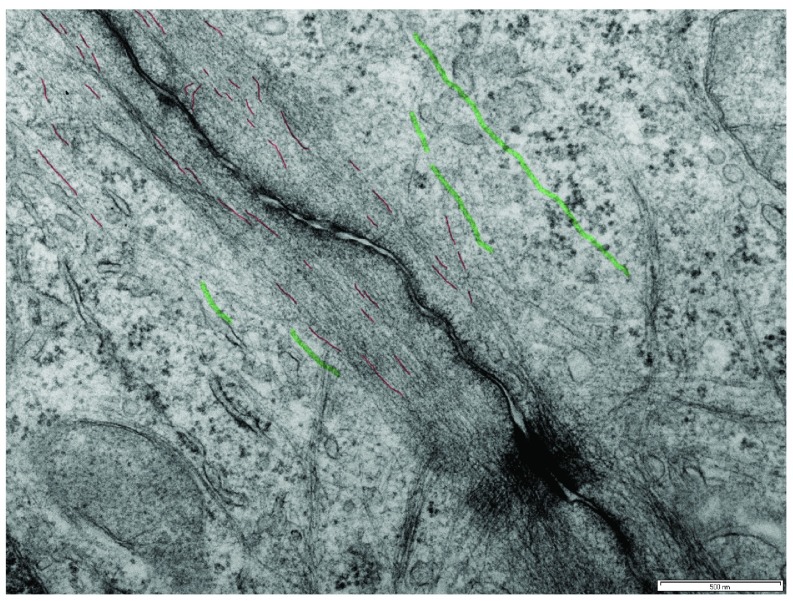
Junctional region between two MCF7 cells as viewed by conventional transmission electron microscopy culture showing the complexity of the membrane-cytoskeletal architecture. Microtubules are highlighted in green, and putative actin filaments in red. Bar = 500 nm.

Conventional electron microscopy, involving chemical fixation and embedding in resin, is still an excellent method for visualization of the membrane and cytoskeletal elements of cell-cell contacts ( Figure 2). However, note that the complexity of the junctional cytoskeleton makes detailed analyses of its organization difficult. This can be resolved by electron tomography, which involves tilting a relatively thick (for example, 300 nm) section and obtaining images at different angles relative to the specimen. This provides not only a 3D view through the depth of the specimen but also far greater resolution, allowing identification and tracing of individual elements. This has been used to great effect in recent studies of the actin organization in cultured cells with actin filaments running parallel to the adherens junction ^31^.

New methods are now providing far greater sample depths and, for the first time, the ability to examine entire cells, large tissue areas, and even entire organisms (albeit the smaller specimens of the animal kingdom). This method, serial blockface scanning electron microscopy (SEM), relies on the imaging of an exposed blockface by SEM in the back-scattered mode ^32^. Material is removed from the blockface, slice by slice using either a knife or a focused ion beam, within the electron microscope, and the exposed blockface is imaged after each slice is removed to generate literally thousands of serial images. Improvements in back-scattered electron detectors now mean that image quality is approaching that of a conventional transmission electron microscope (and the image is contrast-inverted to give a similar appearance). This technique has the potential to provide large-scale information on the way that cells interact in the culture dish but also in a tissue environment, with the capacity to contain numerous cells in a single 3D data set.

The above methods rely on an initial fixation step, usually using chemical fixatives. The latter can be slow and introduce artefacts, and so there has been a move to cryofixation, usually high-pressure freezing. These methods provide excellent preservation of cellular structures and are becoming routine in many laboratories. However, avoiding chemical fixation by cryofixation introduces another problem: how to go from a frozen sample in liquid nitrogen to an embedded specimen that can be sectioned (note that thin samples can avoid this problem, but this is unlikely to be the case for the study of most cell-cell junctions). Cryosectioning of frozen material provides the optimal method to preserve structure, avoiding both fixatives and any staining process. But this is technically demanding, and the retention of cytoplasmic material can actually hinder visualization of cytoskeletal elements. Freeze substitution, the removal of water at low temperature before embedding, offers a simple and, now, very rapid alternative for embedding in resin after freezing ^33^. Freezing of specimens to sectioning can now be completed in one day. Of particular note for studies aiming to correlate real-time light microscopy with electron microscopy is that methods now exist to maintain the fluorescence of green fluorescent protein and related proteins in resin-processed material ^34– 38^. Thus, the behavior of proteins can be followed in real-time, and the cells then fast-frozen to capture a rapid transient event and then processed for embedding in resin. The same material then can be analyzed by light microscopy and by electron microscopy to allow precise correlation of the two sets of observations. Recent modifications of these methods have described fluorescent proteins that are resistant to harsh fixation conditions ^39^, opening the possibility for correlative microscopy to combine super-resolution imaging of fluorescent proteins with electron microscopy to better characterize their local cellular nano-environment.

Ultimately, researchers would like to see and recognize all the components involved in cell-cell interactions and understand their precise molecular arrangement. We can already see and recognize some of those components, such as F-actin and junctions, and, as described above, we can see them in 3D and increasingly even in the context of whole tissues. But what about the recognition of other components? Can we imagine visualizing individual cadherin molecules or the key regulators of the junctional actin network in a quantitative fashion? Immunogold labeling has long been used to label on sections, and this method has been the gold standard for ultrastructural localization studies ^40^. However, immunogold labeling is relatively inefficient and labeling is generally restricted to the surface of the section (and therefore is hardly useful for the 3D methods such as electron tomography and serial blockface SEM). The most efficient method, using thawed frozen sections, provides excellent visualization of membranes ^41^ but is not routinely useful for visualizing cytoskeletal structures. But new labeling methods are offering possibilities for genetic tagging of proteins for electron microscopy. Of these, the most promising appears to be a peroxidase construct which can be fused to any protein of interest ^42^. The expressed fusion protein can be visualized by using a simple peroxidase reaction on the fixed material to deposit an electron-dense precipitate at the site of the fusion protein. This method may appear to lack the precision of a particulate marker, but the enzyme is directly fused to the protein of interest rather than being detected with antibodies. Importantly, the reaction product can also be detected within the depth of a thick section (for tomography) or in a whole cell or tissue sample, facilitating detection of a protein of interest by serial blockface SEM. This has immense potential for 3D studies of protein localization.

## Future directions

We are living in a Golden Age for biological imaging, where new microscopy techniques and reagents are allowing us to identify biological structures with unparalleled detail and to interrogate the chemical and physical properties of cells and tissues. Nor is it likely that we have exhausted the possibilities. Already light sheet microscopy in its developing forms provides the opportunity to analyze whole organisms in a comprehensive, dynamic manner ^43^. One consequence of these advances has been the generation of quantitative data, and this has entailed the need for mathematical and statistical tools to analyze often very large data sets. These large data sets carry challenges for how we present and “consume” such data. It seems likely that this will promote an even greater nexus between theory and experiment in biology. Just as seeing can be believing, so can our pre-existing ideas and beliefs influence what we see. The application of new physical theory provides the opportunity to develop predictive models, which are informed by the new dynamic and quantitative data that microscopy provides and which yield predictions for further experimentation. These new advances in microscopy and theory provide the chance for us to interrogate complex biological phenomena at cell-cell junctions across vastly different length and time scales, from molecular events to organismal development.

## References

[1] Galbraith CG Galbraith JA: Super-resolution microscopy at a glance. *J Cell Sci.* 2011;124(Pt 10):1607–11. 10.1242/jcs.080085 21536831 PMC3085433

[2] Gomez GA McLachlan RW Wu SK: An RPTPα/Src family kinase/Rap1 signaling module recruits myosin IIB to support contractile tension at apical E-cadherin junctions. *Mol Biol Cell.* 2015;26(7):1249–62. 10.1091/mbc.E14-07-1223 25631816 PMC4454173

[3] Truong Quang BA Mani M Markova O: Principles of E-cadherin supramolecular organization *in vivo*. *Curr Biol.* 2013;23(22):2197–207. 10.1016/j.cub.2013.09.015 24184100

[4] Wu Y Kanchanawong P Zaidel-Bar R: Actin-delimited adhesion-independent clustering of E-cadherin forms the nanoscale building blocks of adherens junctions. *Dev Cell.* 2015;32(2):139–54. 10.1016/j.devcel.2014.12.003 25600236

[5] Angres B Barth A Nelson WJ: Mechanism for transition from initial to stable cell-cell adhesion: kinetic analysis of E-cadherin-mediated adhesion using a quantitative adhesion assay. *J Cell Biol.* 1996;134(2):549–57. 10.1083/jcb.134.2.549 8707837 PMC2120882

[6] Yap AS Brieher WM Pruschy M: Lateral clustering of the adhesive ectodomain: a fundamental determinant of cadherin function. *Curr Biol.* 1997;7(5):308–15. 10.1016/S0960-9822(06)00154-0 9133345

[7] Cavey M Rauzi M Lenne PF: A two-tiered mechanism for stabilization and immobilization of E-cadherin. *Nature.* 2008;453(7196):751–6. 10.1038/nature06953 18480755

[8] Wu SK Gomez GA Michael M: Cortical F-actin stabilization generates apical-lateral patterns of junctional contractility that integrate cells into epithelia. *Nat Cell Biol.* 2014;16(2):167–78. 10.1038/ncb2900 24413434

[9] Harrison OJ Jin X Hong S: The extracellular architecture of adherens junctions revealed by crystal structures of type I cadherins. *Structure.* 2011;19(2):244–56. 10.1016/j.str.2010.11.016 21300292 PMC3070544

[10] Brasch J Harrison OJ Honig B: Thinking outside the cell: how cadherins drive adhesion. *Trends Cell Biol.* 2012;22(6):299–310. 10.1016/j.tcb.2012.03.004 22555008 PMC3385655

[11] Yap AS Niessen CM Gumbiner BM: The juxtamembrane region of the cadherin cytoplasmic tail supports lateral clustering, adhesive strengthening, and interaction with p120 ^ctn^. *J Cell Biol.* 1998;141(3):779–89. 10.1083/jcb.141.3.779 9566976 PMC2132752

[12] Iversen L Tu HL Lin WC: Molecular kinetics. Ras activation by SOS: allosteric regulation by altered fluctuation dynamics. *Science.* 2014;345(6192):50–4. 10.1126/science.1250373 24994643 PMC4255705

[13] Saunders TE Pan KZ Angel A: Noise reduction in the intracellular pom1p gradient by a dynamic clustering mechanism. *Dev Cell.* 2012;22(3):558–72. 10.1016/j.devcel.2012.01.001 22342545 PMC3312004

[14] Bray D Levin MD Morton-Firth CJ: Receptor clustering as a cellular mechanism to control sensitivity. *Nature.* 1998;393(6680):85–8. 10.1038/30018 9590695

[15] Maître JL Berthoumieux H Krens SF: Adhesion functions in cell sorting by mechanically coupling the cortices of adhering cells. *Science.* 2012;338(6104):253–6. 10.1126/science.1225399 22923438

[16] Levayer R Lecuit T: Biomechanical regulation of contractility: spatial control and dynamics. *Trends Cell Biol.* 2012;22(2):61–81. 10.1016/j.tcb.2011.10.001 22119497

[17] Michael M Yap AS: The regulation and functional impact of actin assembly at cadherin cell-cell adhesions. *Semin Cell Dev Biol.* 2013;24(4):298–307. 10.1016/j.semcdb.2012.12.004 23333496

[18] Hutson MS Tokutake Y Chang MS: Forces for morphogenesis investigated with laser microsurgery and quantitative modeling. *Science.* 2003;300(5616):145–9. 10.1126/science.1079552 12574496

[19] Fernandez-Gonzalez R Simoes Sde M Röper JC: Myosin II dynamics are regulated by tension in intercalating cells. *Dev Cell.* 2009;17(5):736–43. 10.1016/j.devcel.2009.09.003 19879198 PMC2854079

[20] Martin AC Gelbart M Fernandez-Gonzalez R: Integration of contractile forces during tissue invagination. *J Cell Biol.* 2010;188(5):735–49. 10.1083/jcb.200910099 20194639 PMC2835944

[21] Mayer M Depken M Bois JS: Anisotropies in cortical tension reveal the physical basis of polarizing cortical flows. *Nature.* 2010;467(7315):617–21. 10.1038/nature09376 20852613

[22] Bambardekar K Clément R Blanc O: Direct laser manipulation reveals the mechanics of cell contacts in vivo. *Proc Natl Acad Sci U S A.* 2015;112(5):1416–21. 10.1073/pnas.1418732112 25605934 PMC4321260

[23] Ishihara S Sugimura K: Bayesian inference of force dynamics during morphogenesis. *J Theor Biol.* 2012;313:201–11. 10.1016/j.jtbi.2012.08.017 22939902

[24] Brodland GW Conte V Cranston PG: Video force microscopy reveals the mechanics of ventral furrow invagination in Drosophila. *Proc Natl Acad Sci U S A.* 2010;107(51):22111–6. 10.1073/pnas.1006591107 21127270 PMC3009801

[25] Grashoff C Hoffman BD Brenner MD: Measuring mechanical tension across vinculin reveals regulation of focal adhesion dynamics. *Nature.* 2010;466(7303):263–6. 10.1038/nature09198 20613844 PMC2901888

[26] Conway DE Breckenridge MT Hinde E: Fluid shear stress on endothelial cells modulates mechanical tension across VE-cadherin and PECAM-1. *Curr Biol.* 2013;23(11):1024–30. 10.1016/j.cub.2013.04.049 23684974 PMC3676707

[27] Borghi N Sorokina M Shcherbakova OG: E-cadherin is under constitutive actomyosin-generated tension that is increased at cell-cell contacts upon externally applied stretch. *Proc Natl Acad Sci U S A.* 2012;109(31):12568–73. 10.1073/pnas.1204390109 22802638 PMC3411997

[28] Leerberg JM Gomez GA Verma S: Tension-sensitive actin assembly supports contractility at the epithelial zonula adherens. *Curr Biol.* 2014;24(15):1689–99. 10.1016/j.cub.2014.06.028 25065757 PMC5103636

[29] Bershadsky A Kozlov M Geiger B: Adhesion-mediated mechanosensitivity: a time to experiment, and a time to theorize. *Curr Opin Cell Biol.* 2006;18(5):472–81. 10.1016/j.ceb.2006.08.012 16930976

[30] He B Doubrovinski K Polyakov O: Apical constriction drives tissue-scale hydrodynamic flow to mediate cell elongation. *Nature.* 2014;508(7496):392–6. 10.1038/nature13070 24590071 PMC4111109

[31] Buckley CD Tan J Anderson KL: Cell adhesion. The minimal cadherin-catenin complex binds to actin filaments under force. *Science.* 2014;346(6209):1254211. 10.1126/science.1254211 25359979 PMC4364042

[32] Denk W Horstmann H: Serial block-face scanning electron microscopy to reconstruct three-dimensional tissue nanostructure. *PLoS Biol.* 2004;2(11):e329. 10.1371/journal.pbio.0020329 15514700 PMC524270

[33] McDonald KL Webb RI: Freeze substitution in 3 hours or less. *J Microsc.* 2011;243(3):227–33. 10.1111/j.1365-2818.2011.03526.x 21827481

[34] Kukulski W Schorb M Kaksonen M: Plasma membrane reshaping during endocytosis is revealed by time-resolved electron tomography. *Cell.* 2012;150(3):508–20. 10.1016/j.cell.2012.05.046 22863005

[35] Kukulski W Schorb M Welsch S: Correlated fluorescence and 3D electron microscopy with high sensitivity and spatial precision. *J Cell Biol.* 2011;192(1):111–9. 10.1083/jcb.201009037 21200030 PMC3019550

[36] Kukulski W Schorb M Welsch S: Precise, correlated fluorescence microscopy and electron tomography of lowicryl sections using fluorescent fiducial markers. *Methods Cell Biol.* 2012;111:235–57. 10.1016/B978-0-12-416026-2.00013-3 22857932

[37] Nixon SJ Webb RI Floetenmeyer M: A single method for cryofixation and correlative light, electron microscopy and tomography of zebrafish embryos. *Traffic.* 2009;10(2):131–6. 10.1111/j.1600-0854.2008.00859.x 19054388

[38] Schieber NL Nixon SJ Webb RI: Modern approaches for ultrastructural analysis of the zebrafish embryo. *Methods Cell Biol.* 2010;96:425–42. 10.1016/S0091-679X(10)96018-4 20869533

[39] Paez-Segala MG Sun MG Shtengel G: Fixation-resistant photoactivatable fluorescent proteins for CLEM. *Nat Methods.* 2015;12(3):215–8, 4 p following 218. 10.1038/nmeth.3225 25581799 PMC4344411

[40] Slot JW Geuze HJ: Cryosectioning and immunolabeling. *Nat Protoc.* 2007;2(10):2480–91. 10.1038/nprot.2007.365 17947990

[41] Tokuyasu KT: A technique for ultracryotomy of cell suspensions and tissues. *J Cell Biol.* 1973;57(2):551–65. 10.1083/jcb.57.2.551 4121290 PMC2108989

[42] Martell JD Deerinck TJ Sancak Y: Engineered ascorbate peroxidase as a genetically encoded reporter for electron microscopy. *Nat Biotechnol.* 2012;30(11):1143–8. 10.1038/nbt.2375 23086203 PMC3699407

[43] Chen BC Legant WR Wang K: Lattice light-sheet microscopy: imaging molecules to embryos at high spatiotemporal resolution. *Science.* 2014;346(6208):1257998. 10.1126/science.1257998 25342811 PMC4336192

