# Supplementary material for: Seeing and believing: recent advances in imaging cell-cell interactions
Source: F1000Res. 2015 Jul 17;4(F1000 Faculty Rev):273. [Version 1] doi: 10.12688/f1000research.6435.1 (PMC4623898; doi:10.12688/f1000research.6435.1)
Supplement: Peer review history

# Peer review: Referee response for version 1

**DOI:** 10.5256/f1000research.6903.r9553

**Published:** 2015-07-17

**Authors:** Pakorn T. Kanchanawong

**Affiliations:** 1 Mechanobiology Institute, Department of Biomedical Engineering, National University of Singapore, Singapore, Singapore

I have read this submission. I believe that I have an appropriate level of expertise to confirm that it is of an acceptable scientific standard.

# Peer review: Referee response for version 1

**DOI:** 10.5256/f1000research.6903.r9552

**Published:** 2015-07-17

**Authors:** Keith Burridge

**Affiliations:** 1 University of North Carolina at Chapel Hill, Chapel Hill, NC, USA

I have read this submission. I believe that I have an appropriate level of expertise to confirm that it is of an acceptable scientific standard.

# Peer review: Referee response for version 1

**DOI:** 10.5256/f1000research.6903.r9551

**Published:** 2015-07-17

**Authors:** Pierre-Francois Lenne

**Affiliations:** 1 Institut de Biologie du Développement de Marseille, Aix-Marseille Université, Marseille, France

I have read this submission. I believe that I have an appropriate level of expertise to confirm that it is of an acceptable scientific standard.
